# The Editor's Letter-Box

**Published:** 1921-01-01

**Authors:** Philip A. Inman

**Affiliations:** Secretary Hospital Saturday Fund


*To the Editor of* THE HOSPITAL.

*To the Editor of* THE HOSPITAL.

SIR,—I have read with much interest the correspondence which has appeared under the above heading in recent issues of THE HOSPITAL.

I am in entire agreement with Mr. Glenton-Kerr's statement that " the vast majority of the weekly wage earners in London are already contributing generously to hospital funds." The work of preparing a complete analysis showing the number of business houses where there are hospital collections is one which requires much time and care, but progress has been made, and the result already achieved is both valuable and interesting. A study of the returns already tabulated reveals the following particulars:

48 per cent. of the firms have collections for the Hospital Saturday Fund.

29 per cent. of the firms have collections for the local hospital or a hospital collecting organisation.

10 per cent. of the firms have collections alternatively for the Hospital Saturday Fund and the local hospital.

7 per cent. of the firms have collections for St. Dunstan's, Dr. Barnardo's Homes, and similar societies.

Leaving only 6 per cent. where no collection, so far as can be ascertained, is taken.

One of the most surprising features has been to discover the large number of workpeople's hospital societies; there are no less than nine in the Borough of Bethnal Green alone. These figures prove conclusively that the majority of insured persons already contribute voluntarily to the hospitals and allied institutions, and we must, therefore, seek for increased support in other directions.

I venture to suggest one or two definite ways by which additional help might be obtained.

1. There should be in *every* business house, factory, and workshop a regular weekly collection. In the words of your leading article last week, " there must be no exceptions if the present difficulties are to be met."

It is pleasing to report that the Hospital Saturday Fund has enrolled 150,000 additional subscribers during the current year, and since November 1 280 new collections have been established. I think this will be regarded as satisfactory, especially when one remembers the slump in trade, with its consequent unemployment.

2. The employees in banks, insurance offices, etc., should be urged to co-operate.

I know of a large office which consists of six departments in only one of which is there a hospital collection taken. This is by no means an isolated case. Yet there are indications that the " black-coated " workers are realising their responsibilities in the matter. A few days ago I received the welcome news that a large company had commenced collections on our behalf, and promises amounting to £150 per annum had already been received.

3. Employers should be approached with a view to supplementing the contributions of their employees. There are instances where firms double their workpeople's subscriptions, and others where donations are received in augmentation. When this is done not only does it increase the amount of the collection, but it encourages the employees in their efforts.

4. Wherever practicable collections should be made by means of cards or sheets in lieu of boxes. Collecting-boxes encourage irregular giving.

For some years a well-known city house had what is termed a " Box Collection," and their record subscription amounted to £22. The " Card System " was introduced, the names and contributions of each employee recorded, and the average yearly collection is now £50.

At this critical period in the history of the voluntary hospitals it is incumbent to explore every avenue that will lead to the receipt of increased support, and any suggestion that will help to secure this end is worthy of careful consideration.—I am, yours faithfully,

PHILIP A. INMAN,
Secretary Hospital Saturday Fund.

